# The Effect of Various Fruit Juices Preservation Methods on Their Antioxidant Properties

**DOI:** 10.1155/ijfo/6321214

**Published:** 2026-08-28

**Authors:** Dariusz Nowak, Michał Gośliński, Julia Soja, Magdalena Wudzińska, Emilia Rumińska

**Affiliations:** ^1^ Department of Nutrition and Dietetics, Faculty of Health Sciences, Ludwik Rydygier Collegium Medicum in Bydgoszcz, Nicolaus Copernicus University in Toruń, Bydgoszcz, Poland, umk.pl

**Keywords:** antioxidant properties, *Aronia melanocarpa*, *Mangifera indica*, *Passiflora edulis*, preservation methods, *Vaccinium corymbosum*

## Abstract

Fruit juices constitute a valuable source of vitamins and bioactive compounds exhibiting strong antioxidant properties. The aim of the study was to evaluate the effect of different preservation methods on the antioxidant properties. The materials were: chokeberry (*Aronia melanocarpa*), blueberry (*Vaccinium corymbosum*), passion fruit (*Passiflora edulis*) and mango (*Mangifera indica*). Obtained juices were subjected to six preservation methods: three types of pasteurization (HTST, MTST, MTLT), two types of microwaving (30 and 15‐s) and sonication. One part of each juice was a control sample, not subjected to preservation. The antioxidant capacity (DPPH, ABTS), total polyphenol content (TP, FBBB), total flavonoids (TF), and total anthocyanins (TA) were determined. *A. melanocarpa* juice exhibited the highest polyphenol content and the greatest antioxidant capacity, reaching of ca. 665 and 1020 mg TE/100 mL (DPPH and ABTS, respectively). Juices from *V. corymbosum*, *M. indica*, and *P. edulis* demonstrated lower antioxidant capacity resulting from significantly lower polyphenol content, including flavonoids and anthocyanins. Processing of fruit juices has an impact on the content of their bioactive compounds. Conventional preservation methods based on heating treatment may lead to losses of antioxidant properties, while microwaving and sonication did not reduce them and, in some cases, even enhanced them. In blueberry juice, most of treatments increased antioxidant capacity. In passion fruit juice, the highest activity was observed after HTST and sonication, whereas microwaving and MTLT significantly reduced antioxidant capacity. Overall, all analyzed juices represent valuable sources of bioactive compounds whose properties may be of importance from both nutritional and technological perspectives. Especially *A. melanocarpa* deserves to be regarded as superfruit.

## 1. Introduction

Fruits, vegetables, and the juices obtained from them are a valuable source of vitamins and bioactive compounds with antioxidant properties. Therefore, both berries and many exotic fruits are becoming a subject of research interest. Recent studies indicate that chokeberry and blueberries are rich sources of phytochemicals. Chokeberry originates from the eastern parts of North America and East Canada, and nowadays they are also grown in Central and Eastern Europe. This fruit belongs to the Rosaceae family (Maloideae subfamily). Black chokeberry, also known as *Aronia melanocarpa* (Michx.) Elliott, has significant health benefits. *A. melanocarpa* fruits demonstrate beneficial effects in combating diseases related to oxidative stress, such as cancer and cardiovascular diseases [1, 2]. This is because of the fact that chokeberry fruits are one of the richest sources of polyphenols and anthocyanins [3]. Nowadays, phytochemical investigations have shown that the fruits of *A. melanocarpa* are the abundant source of phenolic compounds, such as procyanidins, anthocyanins, phenolic acids, and their analogs. The predominant polyphenols of chokeberry juices were procyanidins, cyanidin‐3‐O‐galactoside, cyanidin‐3‐arabinoside, chlorogenic acid, and quercetin. [1, 4–6]. Apart from chokeberries, other fruits containing polyphenols are blueberries, especially *Vaccinium corymbosum* L. Blueberry is a fruit‐bearing shrub in the genus *Vaccinium*, which is part of the heath family (Ericaceae). *V. corymbosum* L. is native to North America but is cultivated worldwide. The United States is the leading blueberry producer in the world. Approximately 60% of the crop is sold as fresh and the remaining goes for processing, mainly frozen [7–9]. Blueberries contain numerous bioactive compounds, such as vitamins and polyphenols, particularly anthocyanins [7, 10]. Because of their flavor, exotic fruits are becoming increasingly popular, including mango (*Mangifera indica* L.) and passion fruit (*Passiflora edulis* Sims). *M. indica* L., known as the king of fruits, has an attractive taste and fragrance and high nutritional value [11]. Mango is a member of the Anacardiaceae family, which originated in India and traditionally grows in tropical climates mainly from the Indo‐Malaysian region. Recently, mango production has increased worldwide [12]. Mango pulp is a rich source of various nutrients such as sugars, amino acids, aromatic compounds, pectins, vitamins, and bioactive compounds, mainly carotenoids and polyphenols [11]. The primary polyphenols identified in mango pulp are gallic acid and galloyl‐derived polyphenols, including mono‐galloyl glucose, and gallotannins (hexa‐ to nona‐O‐galloyl‐glucoses) and represent up to 95% of all polyphenols [12, 13]. Minor polyphenols in mango pulp include vanillic, protocatechuic, p‐hydroxybenzoic acid, p‐coumaric, ferulic, cinnamic, caffeic, chlorogenic acid, mangiferin (xanthones), and quercetin derivatives (flavonoids) [12, 14].

Passion fruit belongs to the family Passifloraceae. The main species of this genus are *P. edulis* Sims (purple passion fruit) and the *P. edulis* var. *flavicarpa* (yellow passion fruit). *P. edulis* is native to South America, but currently found on all continents. The main producer of *P. edulis* is Brazil [15]. The passion fruit pulps are sources of phenolic compounds (flavonoids and phenolic acids), carotenoids such as zeaxanthin, *α*‐tocotrienol, *β*‐carotene, and vitamin C [15, 16]. Passion fruit also contains lipids, amino acids and derivatives, organic acids, nucleotides and derivatives, sugars and alkaloids, lignans, and coumarins [17]. The production and preservation of fruit juices allow them to be used for extended periods, from several days to several months, depending on the preservation method. Currently, traditional preservation methods based on classic pasteurization are used, as well as innovative techniques such as microwaving and sonication. However, juice preservation can significantly affect the chemical composition, content of bioactive compounds (including polyphenols) and antioxidant activity of juices [18]. There is a growing number of literature data concerning the effects of different processing methods on fruit juice quality; however, the results are often inconclusive and depend on the type of fruit and the specific technologies used.

Therefore, the aim of the study was to evaluate the effect of various preservation methods on antioxidant properties of selected fruit juices. In this work, following methods were applied: DPPH and ABTS for antioxidant capacity and Folin–Ciocalteu and Fast Blue BB (FBBB) assay for total polyphenol (TP) content, as well as methods determining total flavonoid (TF) content and total anthocyanins (TA).

## 2. Materials and Methods

### 2.1. Materials

Four fruit species were selected as raw materials for the study: chokeberry (*A. melanocarpa* (Michx.) Elliott; cultivar “Nero”), blueberry (*V. corymbosum* L.; cultivar “Duke”), purple passion (*P. edulis* Sims; cultivar “f. edulis”) and mango (*M. indica* L.; cultivar “Tommy Atkins”). Chokeberries and blueberries were sourced from Poland, whereas passion fruits and mangoes originated from South America. The fruits for the study were obtained from a local supplier (fruit and vegetable wholesaler). All samples were purchased in quantities of 5 kg each.

**Figure 1:**
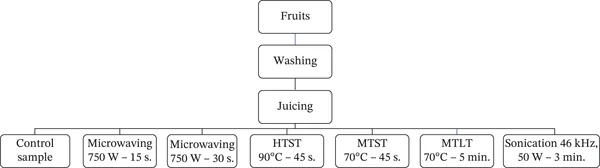
Procedure for handling the tested fruits.

Prior to juicing, the fruits were washed and dried on blotting paper. The fruit juices were extracted using a slow‐speed juicer with a grinding element. Mango (*M. indica* L.) fruits were peeled and pitted before juicing. The seeds of the other fruits were retained in sieves. The juice yield was approximately 600 mL per kilogram of chokeberries and blueberries and approximately 400 mL per kilogram of mangoes and passion fruits. The juices were freshly squeezed and divided into 100 mL glass bottles. Each of the obtained juices was divided into seven parts (Figure 1). Six parts of each juice were subjected to preservation: two types of microwaving at 750 W for 30 and 15 s (MW 30 and MW 15 samples); three types of pasteurization (water bath with controlled parameters of temperature and time): HTST (high temperature short time) at 90°C for 45 s, MTST (medium temperature short time) at 70°C for 45 s), MTLT (medium temperature long time) at 70°C for 5 min (HTST, MTST, and MTLT samples) and sonication (ultrasonic bath at 46 kHz, 50 W) for 3 min (SON sample). One part of each juice was a control sample, not subjected to preservation (CON sample). Before analysis of antioxidant properties the juices were stored briefly in the refrigerator.

### 2.2. Methods

#### 2.2.1. pH Assay

The pH of the samples was determined at room temperature using a glass electrode (Hanna Instruments, Olsztyn, Poland).

#### 2.2.2. Soluble Solids Content

The soluble solids content of samples was measured with a laboratory refractometer RL‐3 (PZO, Warsaw, Poland), at 20°C, following a procedure similar to that described by Rubio‐Arraez et al. [19].

#### 2.2.3. DPPH Assay

The antioxidant capacity of the fruit juices was evaluated using the DPPH radical scavenging assay, based on the method proposed by Yen and Chen with modifications, using 0.1 mmol/L methanol solution of 1,1‐diphenyl‐2‐picrylhydrazyl (DPPH, Sigma‐Aldrich, St. Louis, Missouri, United States) [20]. The DPPH method is commonly applied for the assessment of antioxidant activity in fruits, vegetables, and their juices, whereas its main advantages have been reported in previous studies [21–23]. For the analysis, 0.1 mL of juice sample was combined with 2.9 mL of the DPPH solution and thoroughly mixed. The absorbance was measured on a Rayleigh UV‐1800 V/VIS spectrophotometer at 517 nm after 30 min of incubation at room temperature in the dark. For each juice, samples were analyzed in three replicates and the three measurements were averaged to obtain the final result. The percentage of DPPH scavenging was calculated using Equation (1):

(1) %scavenging=ADPPH–AjuiceADPPH×100,

where A_DPPH_ is the absorbance of the DPPH blank solution and A_juice_ is the absorbance of the sample solution.

The resultant value was then substituted into an equation of a previously prepared 6‐hydroxy‐2,5,7,8‐tetramethylchromane‐2‐carboxylic acid (Trolox–Sigma‐Aldrich) calibration curve. The antioxidant capacity of the samples was expressed as milligrams of Trolox equivalents (Sigma‐Aldrich) per 100 mL of sample.

#### 2.2.4. ABTS Assay

The antioxidant capacity was assessed using the ABTS assay according to the procedure described by Re et al. [24] method with minor modifications. In the ABTS method, 2,2’‐azinobis‐(3‐ethyl‐benzothiazoline‐6‐sulfonic acid) diammonium salt (ABTS, Sigma‐Aldrich, St. Louis, Missouri, United States) and potassium persulfate solutions were mixed and stored overnight at room temperature in the dark for 12–16 h. ABTS solution was diluted with methanol to an absorbance of 0.70 ± 0.02 at 734 nm.

After addition of 1.0 mL of diluted ABTS solution (A734 nm = 0.700 ± 0.020) to 0.01 mL of antioxidant compounds or Trolox standards in methanol, the absorbance was measured on a Rayleigh UV‐1800 V/VIS spectrophotometer at 734 nm against methanol after 1 min. Quantification was performed using a Trolox standard curve. The antioxidant capacity of the samples was expressed as milligrams of Trolox equivalents (Sigma‐Aldrich) per 100 mL of sample.

#### 2.2.5. TP

The TP content of the samples was determined in the Folin–Ciocalteu assay (Sigma‐Aldrich) according to the previously described procedure [25]. For the analysis, 0.3 mL of a sample was transferred in a 10‐mL tube, next followed by the addition of 0.05 mL 2 mol/L Folin–Ciocalteu reagent (Sigma‐Aldrich, St. Louis, Missouri, United States) and 0.5 mL 20% sodium carbonate solution. Subsequently, 4.15 mL of distilled water was added to the reaction mixture, which was then thoroughly mixed. The absorbance was measured on a Rayleigh UV‐1800 V/VIS spectrophotometer at 765 nm after 30 min incubation in the dark at room temperature. A calibration curve was performed with gallic acid. The results were expressed as milligrams of gallic acid equivalents (GAE) per 100 mL of sample.

#### 2.2.6. FBBB Assay

FBBB is a novel method introduced by Medina [26] to quantify the phenolic compounds through direct interaction of polyphenols with the FBBB reagent (4‐benzoylamino‐2,5‐diethoxybenzenediazonium chloride hemi(zinc chloride) salt; Sigma‐Aldrich) under alkaline conditions. According to previous studies, this method demonstrates higher values of GAE than the Folin–Ciocalteu assay does [26, 27]. A 0.2‐mL aliquot of 0.1% FBBB reagent was added to 2 mL of samples and mixed for 1 min and 0.2 mL 5% sodium hydroxide was added. The absorbance was measured on a Rayleigh UV‐1800 V/VIS spectrophotometer at 420 nm after 90 min of incubation in the dark at room temperature. The results are expressed as GAE per 100 mL of sample.

#### 2.2.7. TF Content

The TF content was measured using the colorimetric assay developed by Kapci et al. [22]. For the assay, 0.3 mL of 5% sodium nitrite was added to 1 mL of sample at zero time. After 5 min, 0.3 mL of 10% aluminum chloride was added. At the 6th min, 2 mL of 1 M sodium hydroxide was added. The mixture was diluted by addition of 2.4‐mL distilled water and mixed. The absorbance was measured on a Rayleigh UV‐1800 V/VIS spectrophotometer at 510 nm. The TF content was determined by a (+)‐catechin (Sigma‐Aldrich) standard curve and was expressed as milligrams of catechin (CAE) equivalents per 100 mL of sample.

#### 2.2.8. TA

TA were determined by the pH differential method (AOAC Official Method 2005.02) [28]. Juices were diluted according to appropriate dilution ratios (one part sample and four parts buffer) by adding both 0.025 mol L^−1^ KCl (pH 1.0) or 0.4 mol L^−1^ CH_3_COONa·3H_2_O (pH 4.5) buffer solutions (Avantor Performance Materials). Samples were mixed and left in the dark for 30 min. Absorbance was measured on a Rayleigh UV‐1800 V/VIS spectrophotometer at 520 and 700 nm, and the results were calculated using the following Formula (2):

(2) A=A520700−ApH1.04.5−A520700−ApH,

where A_520_ is the absorbance measured at 520 nm and A_700_ is the absorbance measured at 700 nm, at pH 1.0 and 4.5, respectively.

TA were expressed as milligrams of cyanidin‐3‐mono‐glucoside (CGE) equivalents per 100 mL of sample [28].

Molar extinction coefficient = 26.900 L mol^−1^ mL^−1^ and molecular weight = 449.2 g mol^−1^.

#### 2.2.9. Statistical Analysis

The results were statistically analyzed by calculating the mean and standard deviation. The interpretation of the results was performed with MS Excel Analysis ToolPak software (Microsoft, Redmond, Washington, United States) applying one‐way analysis of variance (ANOVA) and the Tukey′s post hoc test: different letters in the same row or column in the tables indicate statistical significance (at least *p* ≤ 0.05).

## 3. Results and Discussion

Applied preservation methods did not have a significant effect (*p* ≤ 0.05) on the pH values of the analyzed juices (Table 1). Considering the differences among juices, the lowest pH was observed in juice obtained from *P. edulis* fruits. This may be attributed to the relatively high vitamin C content (approx. 30 mg/100 g) in purple passion fruit juice [29]. The pH values of *A. melanocarpa* and *M. indica/V. corymbosum* juices were slightly higher, which may result from a lower vitamin C content or differences in fruit maturity [11, 30, 31]. On the other hand, pH is not only determined by vitamin C content, but also by other compounds, especially organic acids.

**Table 1:** pH—value and soluble solids content results of fruit juices subjected to selected preservation methods.

	pH				Soluble solids content (%)			
Juices	*Aronia melanocarpa*	*Vaccinium corymbosum* L.	*Mangifera indica* L.	*Passiflora edulis*	*Aronia melanocarpa*	*Vaccinium corymbosum* L.	*Mangifera indica* L.	*Passiflora edulis*
CON	4.0 ± 0.1^a^	3.1 ± 0.1^b^	3.9 ± 0.2^a^	2.9 ± 0.1^b^	15.5 ± 0.5^a^	16.0 ± 0.1^a^	12.5 ± 0.1^b^	15.0 ± 0.2^a^
MW30	4.0 ± 0.1^a^	3.2 ± 0.1^b^	3.8 ± 0.2^a^	3.0 ± 0.1^b^	15.5 ± 0.5^a^	16.0 ± 0.1^b^	12.5 ± 0.1^c^	18.5 ± 0.5^a^
MW15	4.0 ± 0.1^a^	3.2 ± 0.1^b^	3.8 ± 0.2^a^	2.9 ± 0.1^b^	15.5 ± 0.5^a^	16.0 ± 0.1^a^	12.5 ± 0.1^b^	16.0 ± 0.2^a^
SON	4.0 ± 0.1^a^	3.2 ± 0.1^b^	3.9 ± 0.2^a^	2.9 ± 0.1^b^	15.5 ± 0.5^a^	16.0 ± 0.1^a^	12.5 ± 0.1^b^	16.0 ± 0.2^a^
HTST	4.0 ± 0.1^a^	3.2 ± 0.1^b^	3.9 ± 0.2^a^	2.9 ± 0.1^b^	15.5 ± 0.5^a^	16.0 ± 0.1^a^	12.5 ± 0.1^b^	16.0 ± 0.2^a^
MTST	4.0 ± 0.1^a^	3.2 ± 0.1^b^	3.8 ± 0.2^a^	2.9 ± 0.1^b^	15.5 ± 0.5^a^	16.0 ± 0.1^a^	12.5 ± 0.1^b^	16.0 ± 0.2^a^
MTLT	4.0 ± 0.1^a^	3.2 ± 0.1^b^	3.8 ± 0.2^a^	2.9 ± 0.1^b^	15.5 ± 0.5^a^	16.0 ± 0.1^b^	12.5 ± 0.1^c^	18.25 ± 0.5^a^

*Note:* CON: control sample; MW30: microwaving 750 W—30 s; MW15: microwaving 750 W—15 s; SON: sonication—46 kHz, 50 W—3 min; HTST: 90°C—45 s, MTST: 70°C—45 s, MTLT: 70°C—5 min. Statistical analysis was performed by one‐way ANOVA using the Tukey′s post hoc test: different letters in the same row indicate statistical significance (*p* ≤ 0.05).

The soluble solids content of the analyzed juices remained unchanged regardless of the preservation method, with the exception of *P. edulis* (samples MW30 and MTLT) (Table 1).

### 3.1. Antioxidant Capacity

Next, the effect of different preservation methods on the antioxidant properties of the analyzed juices has been evaluated. Among the tested juices, *A. melanocarpa* presented the highest antioxidant capacity (DPPH and ABTS assays), reaching 664.9 ± 13.9 and 1019.8 ± 21.9 mg TE/100 mL, respectively (Table 2). The remaining unpreserved juices showed much lower antioxidant capacity, ranging from 31.1 ± 0.2 mg TE/100 mL for *P. edulis* to 106.7 ± 3.4 mg TE/100 mL for *M. indica*. The applied preservation methods did not significantly affect (*p* ≤ 0.05) the antioxidant capacity of *A. melanocarpa* (in both DPPH and ABTS assays). Similar observations were made for *M. indica* juice. In contrast, in the case of *V. corymbosum* and *P. edulis* juices, preservation methods influenced changes in antioxidant capacity. For *V. corymbosum*, with the exception of sonication (SON sample), the applied preservation methods resulted in an increase in antioxidant capacity in both DPPH and ABTS assays. In *P. edulis* juice, the highest antioxidant capacity was observed in samples subjected to HTST pasteurization (90°C, 45 s) and sonication (46 kHz, 50 W, 3 min)—HTST and SON samples. In contrast, microwaving significantly (*p* ≤ 0.05) reduced the antioxidant capacity of *P. edulis* juice, particularly after 30 s (MW30 sample). MTLT pasteurization (70°C, 5 min) also resulted in a statistically significant decrease in the antioxidant capacity of *P. edulis*.

**Table 2:** Antioxidant capacity of various fruit juices subjected to selected preservation methods.

	DPPH (mg TE/100 mL)				ABTS (mg TE/100 mL)			
Juices	*Aronia melanocarpa*	*Vaccinium corymbosum* L.	*Mangifera indica* L.	*Passiflora edulis*	*Aronia melanocarpa*	*Vaccinium corymbosum* L.	*Mangifera indica* L.	*Passiflora edulis*
CON	664.9 ± 13.9^a^	73.7 ± 0.1^d^	106.7 ± 3.4^a^	31.1 ± 0.2^b^	1019.8 ± 21.9^a^	78.6 ± 1.0^d^	113.0 ± 4.6^a^	47.5 ± 1.6^b^
MW30	672.6 ± 17.6^a^	115.0 ± 1.0^a^	107.6 ± 4.1^a^	<1.0^e^	1150.5 ± 70.7^a^	161.8 ± 7.9^a^	113.2 ± 0.9^a^	17.0 ± 0.1^d^
MW15	665.8 ± 18.8^a^	114.8 ± 1.0^a^	100.9 ± 3.0^a^	26.0 ± 0.9^c^	1082.2 ± 44.6^a^	166.7 ± 8.0^a^	116.5 ± 2.6^a^	34.8 ± 0.3^c^
SON	648.4 ± 27.2^a^	67.0 ± 2.3^d^	100.2 ± 3.3^a^	33.5 ± 0.9^b^	1054.0 ± 21.9^a^	81.0 ± 4.6^d^	106.4 ± 6.5^a^	56.4 ± 0.1^a^
HTST	651.0 ± 7.7^a^	95.8 ± 0.8^b^	103.4 ± 8.6^a^	39.7 ± 0.1^a^	1128.3 ± 32.6^a^	125.2 ± 2.7^b^	114.4 ± 7.1^a^	55.9 ± 1.6^a^
MTST	687.3 ± 9.4^a^	98.7 ± 0.5^b^	—	—	1108.1 ± 35.2^a^	128.6 ± 2.9^b^	—	—
MTLT	612.1 ± 15.0^a^	86.0 ± 2.2^c^	99.0 ± 2.2^a^	15.9 ± 0.5^d^	1053.1 ± 16.1^a^	111.8 ± 1.5^c^	103.4 ± 8.9^a^	35.4 ± 0.3^c^

*Note:* CON: control sample; MW30: microwaving 750 W—30 s; MW15: microwaving 750 W—15 s; SON: sonication—46 kHz, 50 W—3 min; HTST: 90°C—45 s; MTST: 70°C—45 s; MTLT: 70°C—5 min. Statistical analysis was performed by one‐way ANOVA using Tukey′s post hoc test: different letters in the same column indicate statistical significance (*p* ≤ 0.05).

Abbreviation: TE, Trolox Equivalents.

### 3.2. Phenolic Compounds

In the subsequent part of the study, the effect of different preservation methods on the content of phenolic compounds was analyzed. Similarly to antioxidant capacity, *A. melanocarpa* showed the highest TP content (Table 3). In the case of this juice, among the applied preservation methods, only sonication (SON sample) resulted in a decrease in TP content. In contrast, samples of *A. melanocarpa* juice subjected to HTST and MTST pasteurization showed the highest T*P* values. For *V. corymbosum*, sonication (SON sample) did not cause changes in polyphenol content compared with the control sample (CON sample). The remaining preservation methods did not reduce TP content. Furthermore, in the case of microwaving (MW30 and MW15 samples) and MTST pasteurization, a significant increase in TP was observed. For *M. indica* juice samples, preservation methods did not affect TP content compared with the control sample. In *P. edulis* juice, similarly to the antioxidant capacity results, microwaving for 30 s (MW30 sample) and MTLT pasteurization at 70°C for 5 min resulted in a significant (*p* ≤ 0.05) reduction in TP content.

**Table 3:** Total polyphenol content of various fruit juices subjected to selected preservation methods.

	TP (mg GAE/100 mL)				FBBB (mg GAE/100 mL)			
Juices	*Aronia melanocarpa*	*Vaccinium corymbosum* L.	*Mangifera indica* L.	*Passiflora edulis*	*Aronia melanocarpa*	*Vaccinium corymbosum* L.	*Mangifera indica* L.	*Passiflora edulis*
CON	1498.8 ± 17.7^b^	209.6 ± 13.9^b^	197.6 ± 10.0^a^	172.8 ± 9.6^a^	2311.1 ± 51.5^b^	399.8 ± 3.6^c^	69.2 ± 0.7^b^	169.0 ± 1.4^b^
MW30	1592.1 ± 23.8^b^	312.5 ± 5.1^a^	218.0 ± 9.2^a^	139.2 ± 22.1^b^	2543.0 ± 39.6^a^	581.6 ± 22.1^a^	78.5 ± 4.2^a^	146.7 ± 2.1^c^
MW15	1539.1 ± 34.1^b^	314.0 ± 2.0^a^	219.4 ± 16.2^a^	187.7 ± 22.5^a^	2532.1 ± 57.4^a^	588.4 ± 18.0^a^	75.1 ± 2.6^a^	210.6 ± 8.0^a^
SON	1360.6 ± 46.7^c^	204.7 ± 3.8^b^	187.4 ± 6.6^a^	180.8 ± 24.7^a^	2288.8 ± 34.5^b^	426.0 ± 10.1^c^	68.3 ± 2.6^b^	203.4 ± 3.5^a^
HTST	1752.2 ± 11.3^a^	315.9 ± 9.9^a^	189.6 ± 4.5^a^	191.6 ± 10.2^a^	2447.5 ± 90.1^ab^	549.2 ± 8.5^a^	80.5 ± 1.4^a^	200.4 ± 2.7^a^
MTST	1767.2 ± 12.9^a^	318.7 ± 8.0^a^	—	—	2506.8 ± 49.7^a^	527.4 ± 7.0^ab^	—	—
MTLT	1591.0 ± 24.2^b^	223.4 ± 4.2^b^	180.7 ± 7.4^a^	136.2 ± 0.3^b^	1802.9 ± 14.7^c^	478.2 ± 18.5^b^	78.4 ± 0.9^a^	156.7 ± 3.3^b^

*Note:* CON: control sample; MW30: microwaving 750 W—30 s; MW15: microwaving 750 W—15 s; SON: sonication—46 kHz, 50 W—3 min; HTST: 90°C—45 s; MTST:70°C—45 s; MTLT: 70°C—5 min. Statistical analysis was performed by one‐way ANOVA using Tukey′s post hoc test: different letters in the same column indicate statistical significance (*p* ≤ 0.05).

The FBBB results showed that sonication (SON sample) did not cause changes in polyphenol content compared with the control sample, with the exception of *P. edulis*, where a significant decrease in polyphenols was observed (Table 3). Similarly, to the Folin‐Ciocalteu method, the two applied microwaving treatments (MW30 and MW15) also resulted in an increase in polyphenol content in the FBBB assay. The same effect was observed in juices subjected to HTST pasteurization.

In the case of *A. melanocarpa*, the high antioxidant capacity (DPPH and ABTS assays) results from its high polyphenol content, including flavonoids and anthocyanins, reaching 798.1 ± 5.3 mg CAE/100 mL and 1284.1 ± 60.4 mg CGE/100 mL, respectively (Table 4). Among the remaining analyzed juices, only *V. corymbosum* juice was characterized by a noticeable TF content and TA. The applied preservation methods did not result in a significant (*p* ≤ 0.05) reduction of TF of any analyzed juices, except *V. corymbosum* juice subjected to MTLT pasteurization. For *A. melanocarpa* juice, an increase of TF was observed in samples preserved by HTST and MTLT pasteurization, as well as by microwaving (MW30), compared with the control sample (CON sample). Microwaving (MW30 and MW15) increased TF in *M. indica* and *P. edulis* juices compared with their respective control samples. In the latter juice, sonication and all pasteurization treatments also increased TF levels. Anthocyanin content of *A. melanocarpa* was quite high and remained unchanged compared with preserved samples (Table 4). Significant fluctuations of TA were observed for *V. corymbosum* juice, with an increasing tendency. Anthocyanins were not detected in *M. indica* or *P. edulis* juices.

**Table 4:** Total flavonoid content and total anthocyanins of various fruit juices subjected to selected preservation methods.

	TF (mg CAE/100 mL)				TA (mg CGE/100 mL)			
Juices	*Aronia melanocarpa*	*Vaccinium corymbosum* L.	*Mangifera indica* L.	*Passiflora edulis*	*Aronia melanocarpa*	*Vaccinium corymbosum* L.	*Mangifera indica* L.	*Passiflora edulis*
CON	798.1 ± 5.3^d^	97.2 ± 3.5^a^	8.9 ± 1.2^b^	6.7 ± 1.2^c^	1284.1 ± 60.4^a^	54.2 ± 2.2^d^	n.d.	n.d.
MW30	828.1 ± 3.2^c^	117.3 ± 8.8^a^	13.6 ± 0.5^a^	17.6 ± 0.8^a^	1235.7 ± 51.3^a^	562.6 ± 24.2^a^	n.d.	n.d.
MW15	793.8 ± 5.6^d^	96.4 ± 4.0^a^	14.9 ± 2.3^a^	12.5 ± 1.0^b^	1241.3 ± 47.4^a^	520.0 ± 8.6^a^	n.d.	n.d.
SON	795.0 ± 2.6^d^	100.5 ± 6.3^a^	9.7 ± 2.4^b^	10.4 ± 1.5^b^	1246.9 ± 42.3^a^	52.0 ± 1.8^d^	n.d.	n.d.
HTST	852.6 ± 6.8^b^	110.6 ± 6.2^a^	8.8 ± 0.1^b^	10.7 ± 1.9^b^	1285.8 ± 63.4^a^	338.4 ± 26.2^b^	n.d.	n.d.
MTST	803.6 ± 6.8^d^	100.2 ± 8.2^a^	—	—	1256.9 ± 38.2^a^	152.8 ± 12.0^c^	n.d.	n.d.
MTLT	869.0 ± 1.6^a^	84.9 ± 4.2^b^	8.0 ± 0.2^b^	9.6 ± 0.7^b^	1313.7 ± 70.1^a^	155.9 ± 8.2^c^	n.d.	n.d.

*Note:* CON: control sample; MW30: microwaving 750 W—30 s; MW15: microwaving 750 W—15 s; SON: sonication—46 kHz, 50 W—3 min; HTST: 90°C—45 s; MTST: 70°C—45 s; MTLT: 70°C—5 min.; n.d. indicates not detected. Statistical analysis was performed by one‐way ANOVA using the Tukey′s post hoc test: different letters in the same column indicate statistical significance (*p* ≤ 0.05).

Furthermore, we determined the correlation between the analytical methods used. The correlation coefficients (*R* ^2^) for antioxidant properties of analyzed juices ranged from 0.953 to 0.997 (Table 5). DPPH and ABTS assays showed a high correlation coefficient (*R* ^2^ = 0.997). In addition, both methods demonstrated a high linear correlation with TP (0.990 and 0.995, respectively), and FBBB (0.978 and 0.982), which confirms that polyphenols contributed more to antioxidant capacity than other compounds. A high correlation between antioxidant capacity (DPPH and ABTS assays) and TF was also demonstrated. A high correlation was also found between TP and FBBB assays and TF (0.993 and 0.984, respectively). Slightly lower correlation coefficients were determined for TA.

**Table 5:** The correlation coefficients (*R* ^2^).

	DPPH	ABTS	TP	FBBB	TF	TA
DPPH		0.997	0.990	0.978	0.989	0.970
ABTS	0.997		0.995	0.982	0.994	0.971
TP	0.990	0.995		0.981	0.993	0.964
FBBB	0.978	0.982	0.981		0.984	0.953
TF	0.989	0.994	0.993	0.984		0.965
TA	0.970	0.971	0.964	0.953	0.965	

Abbreviations: ABTS; 2,2 ^′^‐azino‐di‐[3ethylbenzthiazoline sulphonate]; CAE, catechin equivalents; CGE, cyanidin‐3‐mono‐glucoside equivalents; DPPH, 1,1‐diphenyl‐2‐picrylhydrazyl; FBBB, 4‐benzoylamino‐2,5‐diethoxybenzenediazo‐nium chloride hemi(zinc chloride) salt; GAE, gallic acid equivalents; TA, total anthocyanins; TE, Trolox equivalents; TF, total flavonoid content; TP, total polyphenol content.

Our results showed that among the analyzed fruits, *A. melanocarpa* deserves to be classified as a “superfruit” considering its polyphenol content and antioxidant capacity. The high antioxidant properties of *A. melanocarpa* juice have been confirmed by other researchers. Saracila et al. [32] analyzed the antioxidant capacity and polyphenol content in the leaves, fruits, and pomace of *A. melanocarpa*, highlighting the strong antioxidant properties of different morphological parts of the plant. The lower antioxidant capacity of *V. corymbosum*, *M. indica*, and *P. edulis* juices compared with *A. melanocarpa* juice results from their significantly lower polyphenol content, including flavonoids and anthocyanins.

The high antioxidant capacity and high TP content of *A. melanocarpa* juice (not preserved—control sample) have been confirmed in previous studies. Denev et al. [3] indicated that TP of black chokeberry fruits ranged from 1022 to 1795 mg GAE/100 g. In another research, the TP content in *A. melanocarpa* juice was over 1100 mg GAE/100 mL, which resulted in antioxidant capacity about 500 mg TE/100 g (DPPH assay) [33]. Concluding, these results were consistent with the current study. Zorzi et al. [34] analyzed the antioxidant properties of *V. corymbosum* varieties, which antioxidant capacity determined by DPPH method ranged from 48 to 57 mg TE/100 mL. These values were slightly lower than those obtained in our research (73.7 mg TE/100 mL). In the survey Ercan et al. [35], the TP content in blueberry juice was much lower than in our study—67.2 mg versus 209.6 mg GAE/100 mL. In yet another research, the TP level in blueberry juice was slightly higher and reached 274.7 mg GAE/100 mL [36]. Considering the *M. indica* L. juice, the TP content reached 197.6 mg GAE/100 mL and was slightly higher than that determined by Palafox‐Carlos et al. (120–175 mg GAE/100 g)], whereas the TF content was similar in both studies (8.9 vs. 9.0 mg of catechin equivalents). However, the antioxidant capacity reported by these authors was much higher than in our research [37]. Finally, the TP content of *P. edulis* juice was 172.8 mg GAE/100 mL. Septembre‐Malaterre et al. [38] showed much higher values (286.6 mg GAE/100 g), but these were determined in the fruit. In contrast, the values reported by Sheikh et al. [39] for passion fruit juice were much lower than those obtained in our study, reaching 64.6 mg GAE.

Storage and processing of fruit juices have an impact on the content of vitamins and bioactive compounds. Conventional preservation methods based on heating treatment (e.g., pasteurization) may lead to losses of bioactive compounds. In our study, three types of pasteurization were applied—HTST, MTST, and MTLT—differing in processing parameters such as temperature and treatment time.

Among the applied pasteurization treatments, the lowest antioxidant capacity (DPPH and ABTS assays) was observed in juices heated for the longest time, that is, 5 min, despite the relatively mild temperature of 70°C (MTLT samples). Juices heated for 45 s at 70°C and 90°C exhibited higher antioxidant capacity. Similar observations were made for the TP content, where MTLT‐pasteurized juices also showed the lowest values. In another study, extending pasteurization time (80^°^C ± 2^°^C for 1, 2.5, 5, 10, and 15 min) led to a reduction in bioactive compounds, antioxidant capacity, and vitamin C content in the analyzed fruit juices [40]. The literature data concerning the effects of different pasteurization conditions on antioxidant properties remains inconclusive. One study reported that the antioxidant capacity (DPPH assay) of berry fruit juice decreased by 26% and 27% after pasteurization at 75°C for 15 s and 92°C for 10 s, respectively [41]. In contrast, Chen et al. [42] showed that short‐term heating at high temperature positively affected antioxidant properties of pomegranate juice. Moreover, another study demonstrated that high temperatures pasteurization (95°C for 3 min) increases TP content of blueberry juice [35]. However, pasteurization at high temperature, especially for extended periods, may lead to the degradation of certain polyphenolic compounds, particularly sensitive anthocyanins [43]. Anthocyanins and tannins in berries are susceptible to degradation during processing [44]. However, Gonçalves et al. showed that pasteurization favored vitamin C retention during storage but enhanced the total loss of phenolics without affecting anthocyanin levels [45]. Conventional thermal or novel nonthermal treatments to ensure microbial safety have both positive and negative effects on the anthocyanins. By inactivation of oxidizing enzymes, quantities of anthocyanins may be maintained, but more severe conditions pasteurization may have adverse effects [46].

The above‐mentioned research results mostly indicate that anthocyanin levels decreased with increasing pasteurization time, but there are also studies in which anthocyanin levels remained unchanged. This is similar to our results, in which significant anthocyanin losses were observed in *Vacinnium corymbosum* juice (especially MTLT and MTST pasteurization), whereas there were no significant changes in anthocyanins in *A. melanocarpa* juice. This showed that a longer pasteurization time may cause the destruction of anthocyanins, and on the other hand, high pasteurization temperature and short time (HTST pasteurization) may cause the release of these compounds from the plant matrix and the breakdown of phenolic polymers. Our results clearly indicate that longer pasteurization times (MTLT) reduced TP content (in both TP and FBBB assay) and antioxidant capacity.

In addition to pasteurization, unconventional preservation methods such as microwaving and sonication were applied. Our results showed that mild microwave pasteurization conditions (particularly short exposure time) did not reduce antioxidant capacity or TP content and, in some cases, even enhanced them. Similar observations were reported by Slavov et al. [47], who demonstrated a significant increase in antioxidant activity following mild microwave treatment of beetroot juice. Likewise, Polak et al. showed that microwave pasteurization led to an increase in TP content [48]. Saikia et al. [49] also reported that both microwaved (600 W) and sonicated samples exhibited a positive effect on phenolic content and antioxidant activity. However, another study indicated that nonthermal unconventional preservation methods reduced TP content, anthocyanins, and antioxidant capacity compared with thermal treatments [48]. These observations demonstrate that appropriately selected microwave processing parameters can promote the release of phenolic compounds and increase antioxidant capacity.

In our study, the unconventional preservation method of sonication did not result in significant changes in the antioxidant capacity, TP content, TF content, or anthocyanin content of the analyzed juices compared with the untreated control samples. The various studies showed that sonication (20–100 kHz) is a simple nonthermal technique that operates on the principle of cavitation and has the release of phenolic compounds by disrupting the cell walls of the food matrix [50]. This was confirmed in the studies of Cebulak et al., where sonication contributed to an increase in the content of most of the analyzed polyphenolic compounds in black chokeberry [51]. On the other hand, the influence of sonication on browning, as well as polyphenols and antioxidant activity of fresh apple juice was investigated. It was found that ultrasound can inhibit the browning of fresh apple juice, but decreased the contents of total phenolic content, TF content and reduced the antioxidant activity [52]. In addition to sonication, the literature also reports attempts to apply other unconventional juice preservation methods and to evaluate their impact on the content of bioactive compounds. Polak et al. [48] used unconventional, nonthermal methods of berry fruit juice preservation such as pulsed electric field (PEF) and pulsed light (PL) on selected quality parameters of juices. The authors demonstrated that, compared with thermal methods, nonthermal methods resulted in reduced polyphenol content and antioxidant capacity [48].

Therefore, studies comparing thermal and nonthermal methods are inconclusive. In our study, sonication, compared with other thermal methods, resulted in lower TP values in chokeberry and blueberry juices, whereas no visible changes in these compounds were observed in mango and passion fruit juices. The juices subjected to sonication had relatively high antioxidant capacity compared with thermal methods, with the exception of the blueberry juice.

Future research should also focus on optimizing processing parameters to balance microbial safety with the retention of bioactive compounds and antioxidant capacity. In addition, the stability of these compounds during storage and their bioavailability after consumption warrant further investigation. The stability of polyphenols is determined based on indicators such as degradation time, reaction kinetics, and antioxidant potential, as well as through the analysis of their transformations and degradation products using advanced analytical methods [53]. For example, the bioavailability of fruit‐derived polyphenols was investigated by Castello et al. [54], although the study focused on oranges. It would be worthwhile to extend current research to include the fruits analyzed in the present study, particularly in view of the remarkable antioxidant potential exhibited by chokeberry. Polyphenol bioavailability, however, depends on multiple stages, including their release from the food matrix, digestion, intestinal absorption, microbial transformations, enzymatic modifications, transport within the body, and excretion [55]. Evaluating the effects of combined preservation techniques and scaling these methods for industrial applications may provide valuable insights into their practical feasibility and impact on juice quality.

## 4. Conclusion

Our results showed that among all the analyzed fruits, *A. melanocarpa* deserves to be regarded as a superfruit in terms of polyphenol content and antioxidant capacity. *V. corymbosum, M. indica*, and *P. edulis* juices exhibited lower antioxidant capacity due to their lower content of polyphenols, including flavonoids and anthocyanins. Among the three types of pasteurization (HTST, MTST, and MTLT), differing in processing parameters such as temperature and treatment time, the lowest antioxidant capacity (DPPH and ABTS assays) as well as the lowest TP content were observed in juices heated for the longest time, that is, 5 min, despite the relatively mild temperature of 70°C (MTLT samples). Unconventional preservation methods such as microwaving and sonication had a generally beneficial effect on the antioxidant properties of the analyzed juices. Mild microwave pasteurization conditions (short treatment time) did not reduce antioxidant capacity or TP content and, in some cases, even enhanced them. Sonication did not cause significant changes in antioxidant capacity, TP content, TF content, or anthocyanin content compared with the untreated control samples. Further studies are needed to confirm the results. The application of additional unconventional preservation methods to different types of juices should also be considered. Concluding, obtained results suggest that *A. melanocarpa* juice may serve as a particularly valuable raw material for the production of functional foods or for enriching other fruit juices with antioxidant compounds. Furthermore, it should be emphasized that other analyzed juices, despite their lower antioxidant capacity, also represent a valuable source of bioactive compounds, whose properties may be relevant both nutritional and technological perspectives.

## Author Contributions

Dariusz Nowak: writing—review and editing, writing—original draft, supervision, methodology, investigation, data curation, and conceptualization. Michał Gośliński: writing—review and editing, investigation, and methodology. Julia Soja: writing—review and editing and investigation. Magdalena Wudzińska: methodology and investigation. Emilia Rumińska: methodology and investigation.

## Funding

No funding was received for this manuscript.

## Disclosure

All authors have read and approved the final version of the manuscript, consent to its submission for publication, and agree to be fully accountable for all aspects of the research presented.

## Ethics Statement

This study did not involve human participants or animal experiments. Therefore, ethical approval was not required. All procedures were conducted in accordance with institutional and national research guidelines for studies using plant materials.

## Consent

This research did not involve human subjects or participants requiring informed consent

## Conflicts of Interest

The authors declare no conflicts of interest.

## Data Availability

The data that support the findings of this study are available on request from the corresponding author. The data are not publicly available due to privacy or ethical restrictions.

## References

[1] Ren Y. , Frank T. , Meyer G. , Lei J. , Grebenc J. R. , Slaughter R. , Gao Y. G. , and Kinghorn A. D. , Potential Benefits of Black Chokeberry (*Aronia melanocarpa*) Fruits and Their Constituents in Improving Human Health, Molecules. (2022) 27, no. 22, 10.3390/molecules27227823, 36431924. PMC9696386 36431924

[2] Nowak D. , Kloskowski T. , Gośliński M. , Buhl M. , Wojtowicz E. , Popławski C. , Drewa T. , and Pokrywczyńska M. , Antioxidant Properties of *Aronia melanocarpa* and *Morinda citrifolia* Juices and Their Impact on Bladder Cancer Cell Lines, Medical Science Monitor. (2025) 31, e945120, 10.12659/MSM.945120, 40057821. 40057821 PMC11905606

[3] Denev P. , Kratchanova M. , Petrova I. , Klisurova D. , Georgiev Y. , Ognyanov M. , and Yanakieva I. , Black Chokeberry (*Aronia melanocarpa* (Michx.) Elliot) Fruits and Functional Drinks Differ Significantly in Their Chemical Composition and Antioxidant Activity, Journal of Chemistry. (2018) 2018, no. 1, 10.1155/2018/9574587, 9574587.

[4] Nowak D. , Gośliński M. , and Szwengiel A. , Multidimensional Comparative Analysis of Phenolic Compounds in Organic Juices With High Antioxidant Capacity, Journal of the Science of Food and Agriculture. (2017) 97, no. 8, 2657–2663, 10.1002/jsfa.8089, 27739084. 27739084

[5] Yang S.-Q. , Wang D. , and Gao Y. X. , Gao, Advances in Studies on the Function and Application of *Aronia melanocarpa* , Food Research and Development. (2021) 42, 206–213.

[6] Jurendić T. and Ščetar T. M. , *Aronia melanocarpa* Products and by-Products for Health and Nutrition: A Review, Antioxidants. (2021) 10, no. 7, 10.3390/antiox10071052, 34209985. PMC8300639 34209985

[7] Zhang J. , Li S. , An H. , Zhang X. , and Zhou B. , Integrated Transcriptome and Metabolome Analysis Reveals the Anthocyanin Biosynthesis Mechanisms in Blueberry (*Vaccinium corymbosum* L.) Leaves Under Different Light Qualities, Frontiers in Plant Science. (2022) 13, 1073332, 10.3389/fpls.2022.1073332, 36570935. 36570935 PMC9772006

[8] Siddiq M. and Dolan K. D. , Characterization of Polyphenol Oxidase From Blueberry (*Vaccinium corymbosum* L.), Food Chemistry. (2017) 218, 216–220, 10.1016/j.foodchem.2016.09.061, 27719900. 27719900

[9] Li T. , Wang X. , Elango D. , Zhang W. , Li M. , Zhang F. , Pan Q. , and Wu Y. , Genome-Wide Identification, Phylogenetic and Expression Pattern Analysis of Dof Transcription Factors in Blueberry (*Vaccinium corymbosum* L.), Peer J. (2022) 10, e14087, 10.7717/peerj.14087, 36213501. 36213501 PMC9536302

[10] Kalt W. , Cassidy A. , Howard L. R. , Krikorian R. , Stull A. J. , Tremblay F. , and Zamora-Ros R. , Recent Research on the Health Benefits of Blueberries and Their Anthocyanins, Advances in Nutrition. (2020) 11, no. 2, 224–236, 10.1093/advances/nmz065, 31329250. 31329250 PMC7442370

[11] Lebaka V. R. , Wee Y. J. , Ye W. , and Korivi M. , Nutritional Composition and Bioactive Compounds in Three Different Parts of Mango Fruit, International Journal of Environmental Research and Public Health. (2021) 18, no. 2, 10.3390/ijerph18020741, 33467139. PMC7830918 33467139

[12] Kim H. , Castellon-Chicas M. J. , Arbizu S. , Talcott S. T. , Drury N. L. , Smith S. , and Mertens-Talcott S. U. , Mango (*Mangifera indica* L.) Polyphenols: Anti-Inflammatory Intestinal Microbial Health Benefits, and Associated Mechanisms of Actions, Molecules. (2021) 26, 10.3390/molecules26092732. PMC8124428 34066494

[13] Krenek K. A. , Barnes R. C. , and Talcott S. T. , Phytochemical Composition and Effects of Commercial Enzymes on the Hydrolysis of Gallic Acid Glycosides in Mango (*Mangifera indica* L. cv.‘Keitt’) Pulp, Journal of Agricultural and Food Chemistry. (2014) 62, no. 39, 9515–9521, 10.1021/jf5031554, 25185991. 25185991

[14] Maldonado-Celis M. E. , Yahia E. M. , Bedoya R. , Landázuri P. , Loango N. , Aguillón J. , Restrepo B. , and Guerrero Ospina J. C. , Chemical Composition of Mango (*Mangifera indica* L.) Fruit: Nutritional and Phytochemical Compounds, Frontiers in Plant Science. (2019) 10, 10.3389/fpls.2019.01073, 31681339. PMC6807195 31681339

[15] Pereira Z. C. , Cruz J. M. D. A. , Corrêa R. F. , Sanches E. A. , Campelo P. H. , and Bezerra J. A. , Passion Fruit (*Passiflora* spp.) Pulp: A Review on Bioactive Properties, Health Benefits and Technological Potential, Food Research International. (2023) 166, 112626, 10.1016/j.foodres.2023.112626. 36914332

[16] Asiimwe A. , Kigozi J. B. , Baidhe E. , and Muyonga J. H. , Optimization of Refractance Window Drying Conditions for Passion Fruit Puree, LWT - Food Science and Technology. (2022) 154, 112742, 10.1016/j.lwt.2021.112742. PMC9206970 35734120

[17] Xin M. , Li C. , He X. , Li L. , Yi P. , Tang Y. , Li J. , Liu G. , Sheng J. , and Sun J. , Integrated Metabolomic and Transcriptomic Analyses of Quality Components and Associated Molecular Regulation Mechanisms During Passion Fruit Ripening, Postharvest Biology and Technology. (2021) 180, 10.1016/j.postharvbio.2021.111601, 111601.

[18] Dubrović I. , Herceg Z. , Jambrak A. R. , Badanjak M. , and Dragovi V. , Effect of High Intensity Ultrasound and Pasteurization on Anthocyanin Content in Strawberry Juice, Food Technology and Biotechnology. (2011) 49, 196–204, https://hrcak.srce.hr/file/103539.

[19] Rubio-Arraez S. , Capella J. V. , Castello M. L. , and Ortola M. D. , Physicochemical Characteristics of Citrus Jelly With Non Cariogenic and Functional Sweeteners, Journal of Food Science and Technology. (2016) 53, no. 10, 3642–3650, 10.1007/s13197-016-2319-4, 28017979. 28017979 PMC5147687

[20] Yen G. and Chen H. Y. , Antioxidant Activity of Various Tea Extracts in Relation to Their Antimutagenicity, Journal of Agricultural and Food Chemistry. (1995) 43, no. 1, 27–32, 10.1021/jf00049a007.

[21] Apak R. , Gorinstein S. , Bohm V. , Schaich K. , Ozyurek M. , and Guclu K. , Methods of Measurement and Evaluation of Natural Antioxidant Capacity/Activity (IUPAC Technical Report), Pure and Applied Chemistry. (2013) 85, no. 5, 957–998, 10.1351/PAC-REP-12-07-15.

[22] Kapci B. , Neradova E. , Cizkova H. , Voldrich M. , Rajchl A. , and Capanoglu E. , Investigating the Antioxidant Capacity of Chokeberry (*Aronia melanocarpa*) Products, Journal of Food and Nutrition Research. (2013) 52, 219–229.

[23] Nowak D. and Gośliński M. , Assessment of Antioxidant Properties of Classic Energy Drinks in Comparison With Fruit Energy Drinks, Foods. (2020) 9, no. 1, 10.3390/foods9010056, 31935989. PMC7023211 31935989

[24] Re R. , Pellegrini N. , Proteggente A. , Pannala A. , Yang M. , and Rice-Evans C. , Antioxidant Activity Applying an Improved ABTS Radical Cation Decolorization Assay, Free Radical Biology and Medicine. (1999) 26, no. 9-10, 1231–1237, 10.1016/S0891-5849(98)00315-3, 10381194. 10381194

[25] Singleton V. L. , Orthofer R. , and Lamuela-Raventos R. M. , [14] Analysis of Total Phenols and Other Oxidation Substrates and Antioxidants by Means of Folin-Ciocalteu Reagent, Methods in Enzymology. (1999) 299, 152–178, 10.1016/S0076-6879(99)99017-1.

[26] Medina M. B. , Determination of the Total Phenolics in Juices and Superfruits by a Novel Chemical Method, Journal of Functional Foods. (2011) 3, no. 2, 79–87, 10.1016/j.jff.2011.02.007.

[27] Nowak D. , Kłębukowska L. , and Gośliński M. , Antioxidant Properties and Antibacterial Activity of Selected Herbal Teas, Scientific Reports. (2025) 15, no. 1, 41438, 10.1038/s41598-025-26960-8, 41272026. 41272026 PMC12639085

[28] Lee J. , Durst R. W. , Wrolstad R. E. , Collaborators: , Eisele T. , Giusti M. M. , Hach J. , Hofsommer H. , Koswig S. , Krueger D. A. , Kupina S. , Martin S. K. , Martinsen B. K. , Miller T. C. , Paquette F. , Ryabkova A. , Skrede G. , Trenn U. , and Wightman J. D. , Determination of Total Monomeric Anthocyanin Pigment Content of Fruit Juices, Beverages, Natural Colorants, and Wines by the pH Differential Method: Collaborative Study, Journal of AOAC International. (2005) 88, no. 5, 1269–1278, 10.1093/jaoac/88.5.1269, 16385975. 16385975

[29] He X. , Luan F. , Yang Y. , Wang Z. , Zhao Z. , Fang J. , Wang M. , Zuo M. , and Li Y. , *Passiflora edulis*: An Insight Into Current Researches on Phytochemistry and Pharmacology, Frontiers in Pharmacology. (2020) 11, 10.3389/fphar.2020.00617, 32508631. PMC7251050 32508631

[30] Kulling S. E. and Rawel H. M. , Chokeberry (*Aronia melanocarpa*)—A Review on the Characteristic Components and Potential Health Effects, Planta Medica. (2008) 74, no. 13, 1625–1634, 10.1055/s-0028-1088306, 18937167. 18937167

[31] Hatice D. , Üner B. , Sarıtaş S. , Bolat E. , Yalçıntaş Y. M. , Kalkan A. E. , Eker F. , Akdaşçi E. , Canbolat A. A. , Pekdemir B. , Kurtgöz N. , Eğin E. , Akdağ I. S. , Varol C. C. , Özbilen A. , Sezer F. , Metin Ö. K. , Taşkın K. M. , Karav S. , Proestos C. , Babagil G. E. , Oz E. , Brennan C. , Zeng M. , Kumar M. , Elobeid T. , and Oz F. , Exploring the Potential of Black Chokeberry (*Aronia melanocarpa*) as a Health-Enhancing Agent: A Comprehensive Overview, Journal of Food Biochemistry. (2025) 2025, no. 1, 8899523, 10.1155/jfbc/8899523.

[32] Saracila M. , Untea A. E. , Oancea A. G. , Varzaru I. , and Vlaicu P. A. , Comparative Analysis of Black Chokeberry (*Aronia melanocarpa* L.) Fruit, Leaves, and Pomace for Their Phytochemical Composition, Antioxidant Potential, and Polyphenol Bioaccessibility, Foods. (2024) 13, no. 12, 10.3390/foods13121856, 38928798. PMC11202527 38928798

[33] Nowak D. , Gośliński M. , and Kłębukowska L. , Antioxidant and Antimicrobial Properties of Selected Fruit Juices, Plant Foods for Human Nutrition. (2022) 77, no. 3, 427–435, 10.1007/s11130-022-00983-2, 35829820. 35829820 PMC9463271

[34] Zorzi M. , Gai F. , Medana C. , Aigotti R. , Morello S. , and Peiretti P. G. , Bioactive Compounds and Antioxidant Capacity of Small Berries, Foods. (2020) 9, no. 5, 10.3390/foods9050623, 32414083. PMC7278679 32414083

[35] Ercan O. , Askin B. , and Kucukoner F. , Effect of Two Different Blueberry (*Vaccinium corymbosum* L.) Juice Processing Methods on Polyphenolic and Anthocyanin Contents, International Journal of Innovative Approaches in Agricultural Research. (2025) 9, no. 3, 232–245, 10.29329/ijiaar.2025.1356.6.

[36] Nowak D. , Gośliński M. , Przygoński K. , and Wojtowicz E. , The Antioxidant Properties of Exotic Fruit Juices From Acai, Maqui Berry and Noni Berries, European Food Research and Technology. (2018) 244, no. 11, 1897–1905, 10.1007/s00217-018-3102-8.

[37] Palafox-Carlos H. , Yahia E. , Islas-Osuna M. A. , Gutierrez-Martinez P. , Robles-Sánchez M. , and González-Aguilar G. A. , Effect of Ripeness Stage of Mango Fruit (*Mangifera indica* L., cv. Ataulfo) on Physiological Parameters and Antioxidant Activity, Scientia Horticulturae. (2012) 135, 7–13, 10.1016/j.scienta.2011.11.027.

[38] Septembre-Malaterre A. , Stanislas G. , Douraguia E. , and Gonthier M.-P. , Evaluation of Nutritional and Antioxidant Properties of the Tropical Fruits Banana, Litchi, Mango, Papaya, Passion Fruit and Pineapple Cultivated in Réunion French Island, Food Chemistry. (2016) 212, 225–233, 10.1016/j.foodchem.2016.05.147, 27374527. 27374527

[39] Sheikh K. A. , Noieaid A. , Gadpoca P. , Sermwiwatwong S. , Jafari S. , Kijpatanasilp I. , Worobo R. W. , and Assatarakul K. , Potency of Dimethyl Dicarbonate on the Microbial Inhibition Growth Kinetics, and Quality of Passion Fruit (*Passiflora edulis*) Juice During Refrigerated Storage, Foods. (2024) 13, no. 5, 10.3390/foods13050719, 38472832. PMC10930659 38472832

[40] Mandha J. , Shumoy H. , Matemu A. O. , and Raes K. , Characterization of Fruit Juices and Effect of Pasteurization and Storage Conditions on Their Microbial, Physicochemical, and Nutritional Quality, Food Bioscience. (2023) 51, 102335, 10.1016/j.fbio.2022.102335.

[41] Azofeifa G. , Quesada S. , Pérez A. M. , Vaillant F. , and Michel A. , Pasteurization of Blackberry Juice Preserves Polyphenol-Dependent Inhibition for Lipid Peroxidation and Intracellular Radicals, Journal of Food Composition and Analysis. (2015) 42, 56–62, 10.1016/j.jfca.2015.01.015.

[42] Chen D. , Xi H. , Guo X. , Qin Z. , Pang X. , Hu X. , Liao X. , and Wu J. , Comparative Study of Quality of Cloudy Pomegranate Juice Treated by High Hydrostatic Pressure and High Temperature Short Time, Innovative Food Science & Emerging Technologies. (2013) 19, 85–94, 10.1016/j.ifset.2013.03.003.

[43] Patras A. , Brunton N. P. , O’Donnell C. , and Tiwari B. K. , Effect of Thermal Processing on Anthocyanin Stability in Foods; Mechanisms and Kinetics of Degradation, Trends in Food Science and Technology. (2010) 21, no. 1, 3–11, 10.1016/j.tifs.2009.07.004.

[44] Howard L. R. , Prior R. L. , Liyanage R. , and Lay J. O. , Processing and Storage Effect on Berry Polyphenols: Challenges and Implications for Bioactive Properties, Journal of Agricultural and Food Chemistry. (2012) 60, no. 27, 6678–6693, 10.1021/jf2046575, 22243517. 22243517

[45] Gonçalves G. A. S. , Resende N. S. , Carvalho E. E. N. , Resende J. V. , and Vilas Boas E. V. B. , Effect of Pasteurisation and Freezing Method on Bioactive Compounds and Antioxidant Activity of Strawberry Pulp, International Journal of Food Sciences and Nutrition. (2017) 68, no. 6, 682–694, 10.1080/09637486.2017.1283681, 28139162. 28139162

[46] Weber F. and Larsen L. R. , Influence of Fruit Juice Processing on Anthocyanin Stability, Food Research International. (2017) 100, Part 3, 354–365, 10.1016/j.foodres.2017.06.033, 28964358. 28964358

[47] Slavov A. , Karagyozov V. , Denev P. , Kratchanova M. , and Kratchanov K. , Antioxidant Activity of Red Beet Juices Obtained After Microwave and Thermal Pretreatments, Czech Journal of Food Sciences. (2013) 31, no. 2, 139–147, 10.17221/61/2012-CJFS.

[48] Polak N. , Kalisz S. , Wiktor A. , and Kruszewski B. , The Influence of PEF, Pulsed Light, Microwave and Conventional Heat Treatments on Quality Parameters of Berry Fruit Juice Blends, Applied Sciences. (2025) 15, no. 17, 10.3390/app15179234.

[49] Saikia S. , Mahnot N. K. , and Mahanta C. L. , A Comparative Study on the Effect of Conventional Thermal Pasteurisation, Microwave and Ultrasound Treatments on the Antioxidant Activity of Five Fruit Juices, Food Science and Technology International. (2016) 22, no. 4, 288–301, 10.1177/1082013215596466, 26190045. 26190045

[50] Meena L. , Gowda N. N. , Sunil C. K. , Rawson A. , and Janghu S. , Effect of Ultrasonication on Food Bioactive Compounds and Their Bio-Accessibility: A Review, Journal of Food Composition and Analysis. (2024) 126, 105899, 10.1016/j.jfca.2023.105899.

[51] Cebulak T. , Oszmiański J. , Kapusta I. , and Lachowicz S. , Effect of UV-C Radiation, Ultra-Sonication Electromagnetic Field and Microwaves on Changes in Polyphenolic Compounds in Chokeberry (*Aronia melanocarpa*), Molecules. (2017) 22, no. 7, 10.3390/molecules22071161, 28704941. PMC6152385 28704941

[52] Sun Y. , Zhong L. , Cao L. , Lin W. , and Ye X. , Sonication Inhibited Browning but Decreased Polyphenols Contents and Antioxidant Activity of Fresh Apple (*malus pumila mill, cv. Red Fuji*) Juice, Journal of Food Science and Technology. (2015) 52, no. 12, 8336–8342, 10.1007/s13197-015-1896-y, 26604412. 26604412 PMC4648873

[53] Xiao J. , Recent Advances on the Stability of Dietary Polyphenols, eFood. (2022) 3, no. 3, 10.1002/efd2.21.

[54] Castello F. , Fernández-Pachón M. S. , Cerrillo I. , Escudero-López B. , Ortega A. , Rosi A. , Bresciani L. , Del Rio D. , and Mena P. , Absorption, Metabolism, and Excretion of Orange Juice (poly)Phenols in Humans: The Effect of a Controlled Alcoholic Fermentation, Archives of Biochemistry and Biophysics. (2020) 695, 108627, 10.1016/j.abb.2020.108627, 33039389. 33039389

[55] Bohn T. , Dietary Factors Affecting Polyphenol Bioavailability, Nutrition Reviews. (2014) 72, no. 7, 429–452, 10.1111/nure.12114. 24828476

